# Real-Time PCR Validation for *Mycobacterium tuberculosis* Complex Detection Targeting IS*6110* Directly From Bovine Lymph Nodes

**DOI:** 10.3389/fvets.2021.643111

**Published:** 2021-04-26

**Authors:** José María Sánchez-Carvajal, Ángela Galán-Relaño, Inés Ruedas-Torres, Francisco Jurado-Martos, Fernanda Larenas-Muñoz, Eduardo Vera, Lidia Gómez-Gascón, Fernando Cardoso-Toset, Irene Magdalena Rodríguez-Gómez, Alfonso Maldonado, Librado Carrasco, Carmen Tarradas, Jaime Gómez-Laguna, Inmaculada Luque

**Affiliations:** ^1^Department of Anatomy and Comparative Pathology and Toxicology, University of Córdoba, Córdoba, Spain; ^2^Department of Animal Health, University of Córdoba, Córdoba, Spain; ^3^CICAP—Food Research Centre, Córdoba, Spain

**Keywords:** bovine tuberculosis, fresh lymph node, IS*6110*, direct qPCR, *Mycobacterium tuberculosis* complex

## Abstract

Rapid and accurate diagnostic tools, such as Real-Time PCR (qPCR), need to be implemented as a confirmatory test in the framework of bovine tuberculosis (bTB) surveillance and control programs, shortening the turnaround time to confirm bTB infection. The present study aimed to evaluate a direct qPCR from fresh tissue samples targeting the insertion sequence IS*6110* using individually homogenized bovine lymph nodes compared with microbiological culture. Retropharyngeal, tracheobronchial, and mesenteric lymph nodes fresh tissue samples (*n* = 687) were collected from 230 different cattle carcasses at the slaughterhouse. Only 23 of the 230 examined animals showed tuberculosis-like lesions, with 62 of 230 considered as positive. Among these 62 animals, 61 resulted as culture-positive, whereas 48 were qPCR-positive. Thus, this qPCR targeting IS*6110* showed an apparent diagnostic sensitivity and specificity values of 77.1% [95% confidence interval (CI): 66.5–87.6%] and 99.4% (95% CI: 98.3–100.6%), respectively, and a positive predictive value of 97.9% (95% CI: 93.9–102.0%) and negative predictive value of 92.3% (95% CI: 88.4–96.2%). Positive and negative likelihood ratios were 130.2 and 0.2, respectively, and the agreement between microbiological culture and this qPCR was almost perfect (κ = 0.82). These results highlight this qPCR targeting IS*6110* as a suitable complementary method to confirm bTB in animals with either tuberculosis-like lesions or non-tuberculosis-like lesions, decreasing the number of samples subjected to microbiological culture and, hence, its overall associated costs and the turnaround time (under 48 h) to confirm bTB infection. Besides, sampling mesenteric lymph node, which is uncommonly sampled, together with tracheobronchial and retropharyngeal ones, is advisable during postmortem inspection in bTB surveillance programs at the slaughterhouse, especially in areas with a low bTB prevalence scenario.

## Introduction

Bovine tuberculosis (bTB) is a chronic infectious disease caused by *Mycobacterium bovis* and other members of the *Mycobacterium tuberculosis* complex (MTC) (1, 2) that affects various species of mammals, including humans (3, 4). bTB is still one of the largely neglected zoonotic diseases, particularly in developing countries, as the control and surveillance programs for this disease are inadequate or are not carried out, and domestic and wild animals, which act as reservoirs, often share pasture areas. Thereby, it has been estimated that a quarter of the world's population has latent tuberculosis, requiring a global effort to develop new tools for the diagnostic and treatment of this disease (5). In the European Union (EU), bTB primarily affects livestock, which is of economic importance due to its impact on trade. Indeed, bTB is subjected to national eradication programs based on skin testing of all registered cattle herds, slaughtered policy, and abattoir surveillance (Council Directive 64/432/EEC). According to the EU legislation, the official diagnosis of bTB is based on the detection of the cellular immune response (single intradermal tuberculin testing) in reactor animals (skin test-positive animals), which is followed by slaughtering, histopathological examination of atypical or enlarged lymph nodes or parenchymatous organs with tuberculosis-like lesions (TBLs), and/or culture of MTC in primary isolation medium (6). Although a substantial economic expenditure is addressed to ensure efficient surveillance systems and control programs, the detection and confirmation of bTB infection in cattle herds should be more reliable and swifter (7).

Microbiological culture is considered the reference technique for bTB diagnosis with recovery rates ranging from 30 to 95% (8–10) and sensitivity (SE) and specificity (SP) values of 78.1 and 99.1%, respectively (7). It is reported that culture is an imperfect, laborious, and time-consuming technique that requires high biosecurity facilities and relatively high expertise (7, 8), whose performance can, moreover, be affected by several factors (8–11). A major drawback is the delayed culturing process (up to 2–3 months), making the time required to reach a final diagnosis longer (7, 11, 12).

In the current landscape, rapid, cost-effective, and accurate diagnostic tools could pave the way for managing and controlling bTB in cattle herds (13). Although enzyme-linked immunosorbent assay testing is useful to detect anergic tuberculous cattle as a complement to single intradermal tuberculin testing, this assay is not routinely applied in bTB control programs because of its reduced SE (14–16). By contrast, real-time PCR assays [so-called quantitative PCR (qPCR)] have been shown to directly detect MTC in fresh bovine tissue samples with moderate to high estimates of SE and SP (7, 11, 16). Direct qPCR can detect small amounts of MTC DNA independently of its viability with a turnaround time of 24–48 h, shortening the required time to reach confirmatory results (7, 15, 16).

IS*6110* is a target sequence with multiple copies only present in pathogens belonging to MTC, commonly used for MTC detection by PCR (7, 12, 17, 18). Besides IS*6110*, other targets have also been used for the same purpose, including IS*1081* (16, 19), hupB (20, 21), 16S-23S rRNA internally transcribed spacer (22), p34 gene (23), TbD1 (24), or mpb70 (11) with varying results.

In the light of the earlier mentioned, rapid and accurate diagnostic tools, such as qPCR, may be implemented as a confirmatory test in the framework of bTB surveillance and control programs at the slaughterhouse to shorten turnaround time and inform decision-makers on time. Therefore, the present study firstly aimed to evaluate the diagnostic performance of a direct qPCR from fresh tissue samples targeting IS*6110* using individually homogenized lymph nodes and, secondly, to validate the IS*6110* qPCR for the detection of MTC positive samples and animals in the framework of the bTB eradication campaign.

## Materials and Methods

### Samples Selection and Processing

Fresh retropharyngeal, tracheobronchial, and mesenteric lymph node tissue samples (*n* = 687) were collected from 230 cattle carcasses at the slaughterhouse from 2018 to 2019. All samples were collected during routine postmortem veterinary examination within an official context and agreeing with national and European regulations. No purpose killing of animals was performed for this study, so no ethical or farmer's consent approval was required.

Every lymph node was independently sliced, and the presence of visible-TBL or non-tuberculosis-like lesions (NTBLs) was recorded. Individual homogenization was carried out to obtain a uniform mixture of every lymph node independently using a tissue homogenizer (Fisherbrand, Fisher Scientific, Madrid, Spain). Briefly, 4–7 g of each lymph node tissue was placed into a 15-ml Falcon^TM^ tube (Corning, Madrid, Spain) with the same volume (w/v: 1/1) of 0.85% sterile sodium chloride and ground until a homogeneous mixture was obtained. Tissue homogenate was used for DNA isolation and selective bacterial culture.

### *Mycobacterium tuberculosis* Complex Microbiological Culture

Selective bacterial culture was performed in the BSL3 facilities of the Production and Animal Health Laboratory of Córdoba (LPSACo, Regional Government of Andalusia). Briefly, the homogenate was decontaminated with an equal volume of 0.75% (w/v: 1/1) hexadecyl pyridinium chloride solution in agitation for 30 min (25). Samples were centrifuged for 30 min at 1,500×*g*. The pellets were collected with swabs and cultured in liquid media (MGIT^TM^ 960, Becton Dickinson, Madrid, Spain) using an automatized BD Bacter^TM^ MGIT^TM^ System (Becton Dickinson). The culture was considered positive when isolates were confirmed as MTC by qPCR (26).

### DNA Extraction From Homogenized Lymph Nodes

DNA extraction from homogenized tissue samples was performed using DNA Extract VK (Vacunek, Bizkaia, Spain) according to the manufacturer's guidelines with several modifications. In brief, a mix of 300 mg of homogenate, 250 μl of sterile distilled water, and 250 μl of sample lysis buffer VK-SB were added in a 2-ml tube containing 300 mg of 0.5-mm glass beads and submitted to mechanical disruption at 30 Hz during 20 min. Then, the lysed tissue was centrifuged for 5 min at 7,000×*g*, transferring 200 μl of supernatant to a new 1.5-ml tube. Enzymatic digestion was carried out with 25 μl of 20 mg/ml proteinase K at 56°C for 3 h in a thermo-shaker at 750 rpm. After that, 200 μl of the lysis buffer VK-LB3 were added, and the mixture was incubated for 10 min at 70°C. Finally, 210-μl ethanol (96–100%) was added to the sample that was applied in a spin column following the manufacturer's guidelines. DNA elution was run using 100 μl of Tris/hydrochloride buffer supplied with the kit pre-heated at 70°C. Positive and negative extraction controls were also included. All the DNA extraction products were stored at −20°C until use.

### Quantitative Real-Time PCR From Fresh Tissue Samples

The transposon IS*6110*, which is present in all species of the MTC, was the target of this qPCR. Specific primers (IS*6110*-forward: 5′-GGTAGCAGACCTCACCTATGTGT-3′; IS*6110*-reverse: 5′-AGGCGTCGGTGACAAAGG-3′) and a probe (IS*6110*- probe: 5′-FAM-CACGTAGGCGAACCC-MGBNFQ-3′) targeting a conserved region of IS*6110* transposon were used (27). The diagnostic performance of the qPCR was conducted using the QuantiFast® Pathogen PCR + IC Kit (QIAGEN, Hilden, Germany). Amplifications were run in duplicate for each sample in the MyiQ™2 Two-Color qPCR Detection System (Bio-Rad, Hercules, CA, USA) under the following cycling conditions: 95°C for 5 min followed by 45 cycles of 95°C for 15 s and 60°C for 30 s. Following the manufacturer's guidelines, an exogenous inhibition heterologous control [internal amplification control (IAC)] supplied with the kit was included. An inter-run calibrator with a known Ct value of 32 was introduced in each assay to self-control intra-assay repeatably and accuracy. Complete inhibition of amplification was considered when IAC did not amplify and partial inhibition when it showed a cycle threshold (Ct) > 33. When any inhibition was detected, samples were diluted up to a final concentration of 450 ng/μl, and qPCR was run again. Serial 10-fold dilution series of *M. bovis* genomic DNA with known quantities, ranging from 10^6^ to 10^0^, were used as standards to estimate the limit of detection (LOD) or analytical SE. The reactions were carried out in triplicate per dilution in three different assays, and LOD was determined as the lowest concentration in which 95% of replicates were positive according to the Clinical and Laboratory Standards Institute guidelines.

In the case of culture-positive and qPCR-negative samples, DNA extraction and qPCR were repeated to verify the results. Then, proteinase K digestion was increased up to 12 h (overnight incubation) at 56°C in a thermo-shaker at 750 rpm. Positive (MTC confirmed sample) and negative (MTC negative sample) controls were included, as well as an inter-run calibrator. The IS*6110* PCR product of culture-negative and PCR-positive samples were EtOH precipitated, purified using ExoSAP-ITTM (Thermo Fisher Scientific, Barcelona, Spain), and further analyzed by Sanger sequencing (performed at STABvida, Lisbon, Portugal). The obtained sequences were studied using the Bioedit software version 7.1.3.0. Samples confirmed by sequencing were considered as true positives and used to recalculate the diagnostic parameters of the qPCR targeting IS*6110*.

### Validation of Diagnostic Tests

The results of qPCR targeting IS*6110* were compared with microbiological culture ones (*gold standard*) to estimate the diagnostic SE and SP, positive and negative predictive values (PPV and NPV, respectively), and positive and negative likelihood ratios (PLR and NLR, respectively) (WinEpi software 2.0, Faculty of Veterinary Medicine, University of Zaragoza, Spain). Moreover, an agreement between culture and qPCR results was assessed using Cohen's kappa coefficient (κ) (values ≤ 0 indicated no agreement and 0.01–0.20 as none to slight, 0.21–0.40 as fair, 0.41–0.60 as moderate, 0.61–0.80 as substantial, and 0.81–1.00 as almost perfect agreement) (WinEpi software 2.0).

## Results

### Topographical Distribution of Tuberculosis-Like Lesion

A total of 687 retropharyngeal, tracheobronchial, and mesenteric lymph node samples belonging to 230 cattle carcasses were analyzed to serve as evidence of the presence of MTC using microbiological culture and qPCR directly from lymph nodes. Due to the logistic of the slaughterhouse and the timing of slaughtering, it was not always possible to collect the three lymph node samples from all 230 carcasses, lacking one retropharyngeal and two mesenteric lymph nodes. Before being analyzed, every single tissue sample was subjected to a visual inspection to disclose gross lesions, with 26 of 26/687 (3.8%) tissue samples belonging to 23 different cattle (23/230, 10.0%) showing TBL (Table 1). Most of the lesions were evidenced in only one lymph node (tracheobronchial = 11; retropharyngeal = 9), whereas in three animals, TBLs were observed in two lymph nodes (tracheobronchial–retropharyngeal = 1; tracheobronchial–mesenteric = 1; retropharyngeal–mesenteric = 1).

**Table 1:** Evaluation of the microbiological culture and direct qPCR targeting IS*6110* results obtained upon analyzing 687 lymph nodes, according to the presence or absence of tuberculosis-like lesions.

		**TBL**				**NTBL**			
		**RF**	**TB**	**MS**	**Total**	**RF**	**TB**	**MS**	**Total**
Culture	+	5	13	2	20	17	29	7	53
	–	6	0	0	6	201	188	219	608
qPCR	+	8	13	2	23	9 (10)	22 (24)	3	37
	–	3	0	0	3	209 (208)	195 (193)	223	624
Total		11	13	2	26	218	217	226	661

*+, Positive; –, Negative; qPCR, real-time PCR; TBL, tuberculosis-like lesion; NTBL, non-tuberculosis-like lesion; RF, retropharyngeal lymph nodes (n = 229); TB, tracheobronchial lymph node (n = 230) s; MS, mesenteric lymph nodes (n = 228)*.

*Culture-positive and qPCR-negative samples that were finally positive and/or negative to qPCR after DNA extraction was repeated (in brackets)*.

### *Mycobacterium tuberculosis* Complex Microbiological Culture Results

Seventy-three of 687 tissue samples (10.6%) were positive to microbiological culture, whereas 614 were negative (89.4%). Bacteria were detected in tracheobronchial lymph nodes (42 of 73; 57.5%), followed by retropharyngeal (22 of 73; 30.1%), and mesenteric ones (9 of 73; 12.3%) (Table 1). An animal was considered culture-positive when MTC was detected by culture in at least one lymph node. Thus, 61 of 230 animals (26.5%) were positive to culture, whereas 169 were negative (73.5%). MTC was detected in most of the animals (50, 82.0%) in one lymph node (tracheobronchial = 31; retropharyngeal = 16; mesenteric = 3), whereas in 10 animals (16.4%), MTC was detected in two lymph nodes (retropharyngeal–tracheobronchial = 5; tracheobronchial–mesenteric = 5) and in one animal in the three lymph nodes.

### Quantitative Real-Time PCR Targeting IS*6110*

**Figure 1:**
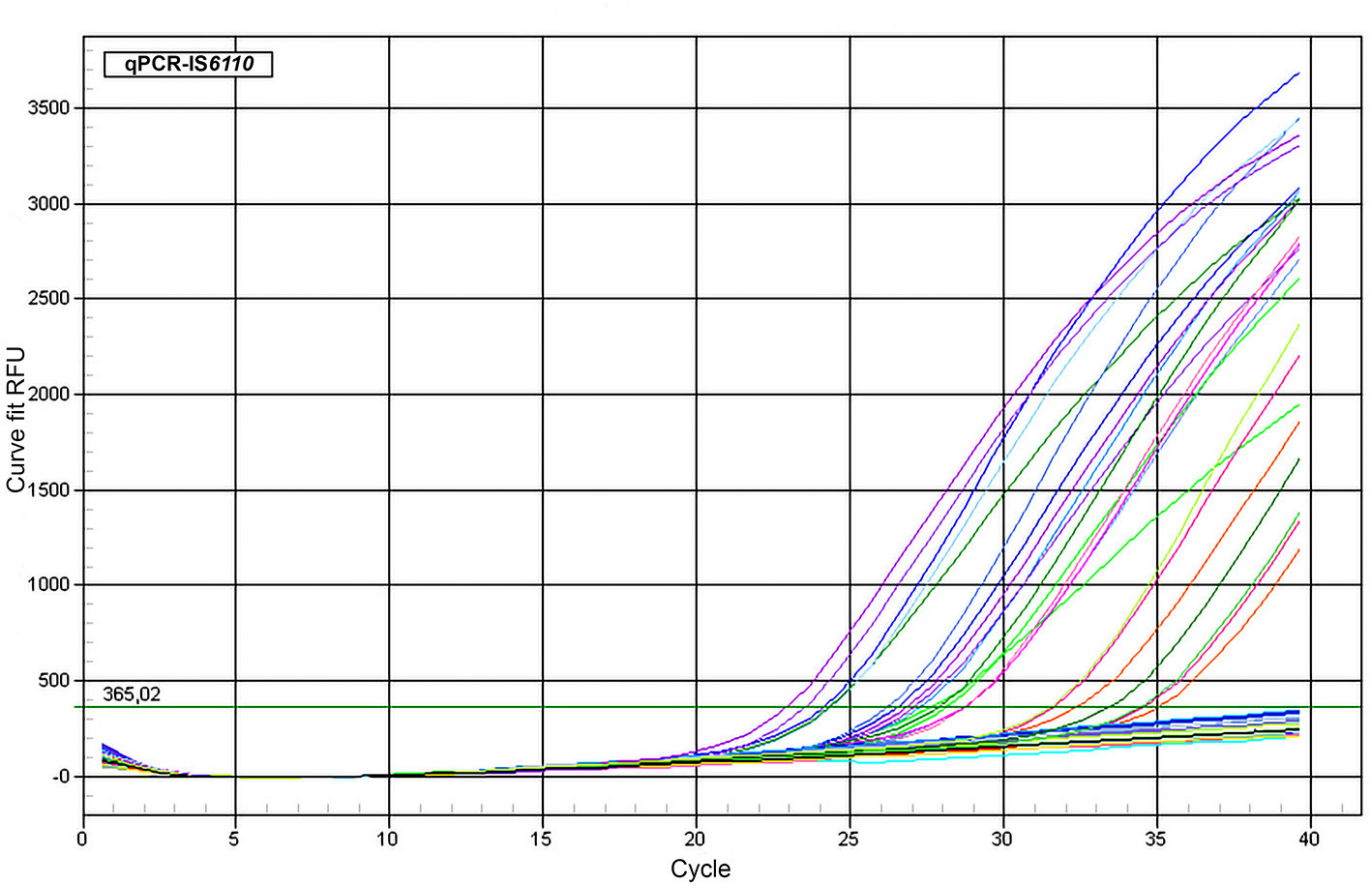
Amplification plot of representative samples. qPCR targeting IS*6110* assay using representative fresh lymph node tissue samples belonging to different cattle. ΔRFU (Y axis) of the reaction was plotted against the Ct value (X axis). Samples with lowest DNA concentration could be detected ranging from 34 to 36 cycles roughly.

Fifty-seven of 687 tissue samples (8.3%) were detected as positive by means of qPCR targeting IS*6110*, with Ct values ranging from 24.2 to 37.5 (Figure 1). The IAC amplified in most of the samples without partial inhibition, showing complete inhibition in eight of the 687 samples due to the high yield of DNA (over 1,000 ng/μl). These samples were diluted up to a final concentration of 450 ng/μl and re-evaluated by qPCR, keeping a negative result for all of them for MTC but with IAC amplification. The LOD for this qPCR-IS*6110* was determined to be ranging from 10 to 100 genomic equivalents, and the cutoff was established to Ct <38. Most of the qPCR-positive results were obtained from tracheobronchial lymph node (35 of 57; 61.4%), followed by retropharyngeal (17 of 57; 29.8%), and mesenteric (five of 57; 8.8%) lymph nodes, reflecting the same trend as observed in the microbiological culture (Table 1).

Following the same criterion used for the microbiological culture, an animal was considered PCR-positive when at least one of the examined lymph nodes yielded a positive result to the qPCR. Thus, 44 of 230 cattle (19.1%) were qPCR-positive, whereas 185 were negative (80.4%). Briefly, 36 of the 44 qPCR positive animals (81.8%) were detected in only one lymph node (tracheobronchial = 24; retropharyngeal = 11; mesenteric = 1), seven animals (15.9%) in two lymph nodes (retropharyngeal–tracheobronchial = 5; tracheobronchial—mesenteric = 2), and one animal (2.3%) in the three lymph nodes.

### Diagnostic Performance of Quantitative Real-Time PCR Compared With Microbiological Culture

Fifty-three of the 73 lymph node samples positive to culture were also positive to qPCR targeting IS*6110*. Because extraction is a rate-limiting factor determining the success of downstream bTB detection by PCR, this step was repeated in those culture-positive and qPCR-negative samples (20/73 samples, 27.4%) to verify the results. Thereby, previous extraction conditions were changed by a proteinase K digestion up to 12 h at 56°C in a thermo-shaker at 750 rpm (overnight incubation), obtaining this time three positive samples of the 20 and remaining the rest (*n* = 17) negative to qPCR. Hence, an apparent SE of 76.7% (95% CI: 67–86.4%) was found. On the other hand, only four of the 614 samples negative to culture were positive to qPCR, with the remaining samples also giving a negative result to qPCR, with an apparent SP value of 99.3% (95% CI: 98.7–100%). The PPV and NPV values were 93.3% (95% CI: 87.0–99.6%) and 97.3% (95% CI: 96.0–98.6%), respectively. In addition, the PLR and NLR were 117.8 and 0.23, respectively. Finally, the concordance or level of agreement between both diagnostic assays for tissue samples was substantial (κ = 0.83) (Tables 1, 2).

**Table 2:** Diagnostic performance of direct qPCR targeting IS*6110* compared with microbiological culture as gold standard analyzing 687 lymph nodes belonging to 230 cattle.

			**True positives**			**Measures of diagnostic accuracy (95% CI)**			
		**Result**	**+**	**−**	**Total**	**Sensitivity**	**Specificity**	**Reliability**	***k* value**
Lymph nodes	qPCR	+	56	4	60	76.7% (67–86.4%)	99.3% (98.7–100%)	96.9% (95.7–98.2%)	0.83
		–	17	610	627				
		Total	73	614	687				
Animals	qPCR	+	47	1	48	77.1% (66.5-87.6%)	99.4% (98.3%-100.6%)	93.5% (90.3–96.7%)	0.82
		–	14	168	182				
		Total	61	169	230				

*+, Positive; –, Negative; qPCR, real-time PCR; 95% CI, 95% confidence level*.

Considering this re-run of the extraction step, 47 of 61 MTC culture-positive animals were also positive for qPCR targeting IS*6110*, resulting in an apparent SE of 77.0% (95% CI: 66.5–87.6%). Only one of the 169 MTC culture-negative animals was positive to qPCR, finding an apparent SP of 99.4% (95% CI: 98.3–100.6%). The measures of PPV and NPV were 97.9% (95% CI: 93.9–102.0%) and 92.3% (95% CI: 88.4–96.2%), respectively. The PLR and NLR were 130.2 and 0.23, respectively. The agreement between microbiological culture and qPCR at the animal level was almost perfect (κ = 0.82) (Table 2).

### Validation of IS*6110* Quantitative Real-Time PCR for the Detection of *Mycobacterium tuberculosis* Complex

Because microbiological culture is considered an imperfect test for bTB diagnosis in which SE may be affected by several factors (7, 8, 16), the combination of culture and IS*6110* qPCR was validated to detect MTC positive samples or animals. In this sense, culture-negative and PCR-positive samples obtained in our study could be considered as MTC positives. This way, the four IS*6110* qPCR-positive and culture-negative lymph node samples were further subjected to Sanger sequencing, and the presence of MTC DNA was evidenced in all of them. Consequently, the diagnostic estimates of the direct qPCR for MTC detection were evaluated, considering as MTC-corrected positive samples, culture-positive samples, and those in which MTC was revealed by Sanger sequencing. For tissue samples, 60 of the 77 MTC-corrected positive samples were successfully amplified by means of qPCR targeting IS*6110* with a corrected SE of 77.9% (95% CI: 68.7–87.2%), SP of 100% (95% CI: 100–100%), and reliability of 97.5% (95% CI: 96.4–98.7%). The PPV and NPV values were increased to 100% (95% CI: 100–100%) and 97.3% (95% CI: 96–98.6%), respectively. The PLR and NLR were 160 and 0.22, respectively, with a level of agreement between assays almost perfect (κ = 0.86) (Table 3).

**Table 3:** Validation of direct qPCR targeting IS*6110* for the detection of MTC analyzing 687 lymph nodes belonging to 230 cattle.

**MTC corrected positive results Culture/Sanger sequencing**						**Measures of diagnostic accuracy (95% CI)**			
		**Result**	**+**	**–**	**Total**	**Sensitivity**	**Specificity**	**Reliability**	***k* value**
Lymph nodes	qPCR	+	60	0	60	77.9% (68.7–87.2%)	100% (100%)	97.5% (96.4–98.7%)	0.86
		–	17	610	627				
		Total	77	610	687				
Animals	qPCR	+	48	0	48	77.4% (67.0–87.8%)	100% (100%)	94% (90.8–97.0%)	0.83
		–	14	168	182				
		Total	62	168	230				

*+, Positive; –, Negative; qPCR, real-time PCR; 95% CI, 95% confidence level*.

*MTC-corrected positive results: (i) samples positive to culture and (ii) culture-negative samples and PCR-positive after Sanger sequencing*.

At the animal level, 48 of the 62 MTC-corrected positive animals were also positive for qPCR targeting IS*6110* with a corrected SE and SP of 77.4% (95% CI: 67–87.8%) and 100% (95% CI: 100–100%), respectively, and reliability of 93.9% (90.8–97.0%). The measures of PPV and NPV were 100% (95% CI: 100–100%) and 92.3% (95% CI: 88.4–96.2%). The PLR and NLR were 100.2 and 0.2, respectively, and the κ value was 0.83 (almost perfect agreement of both assays) (Table 3).

**Figure 2:**
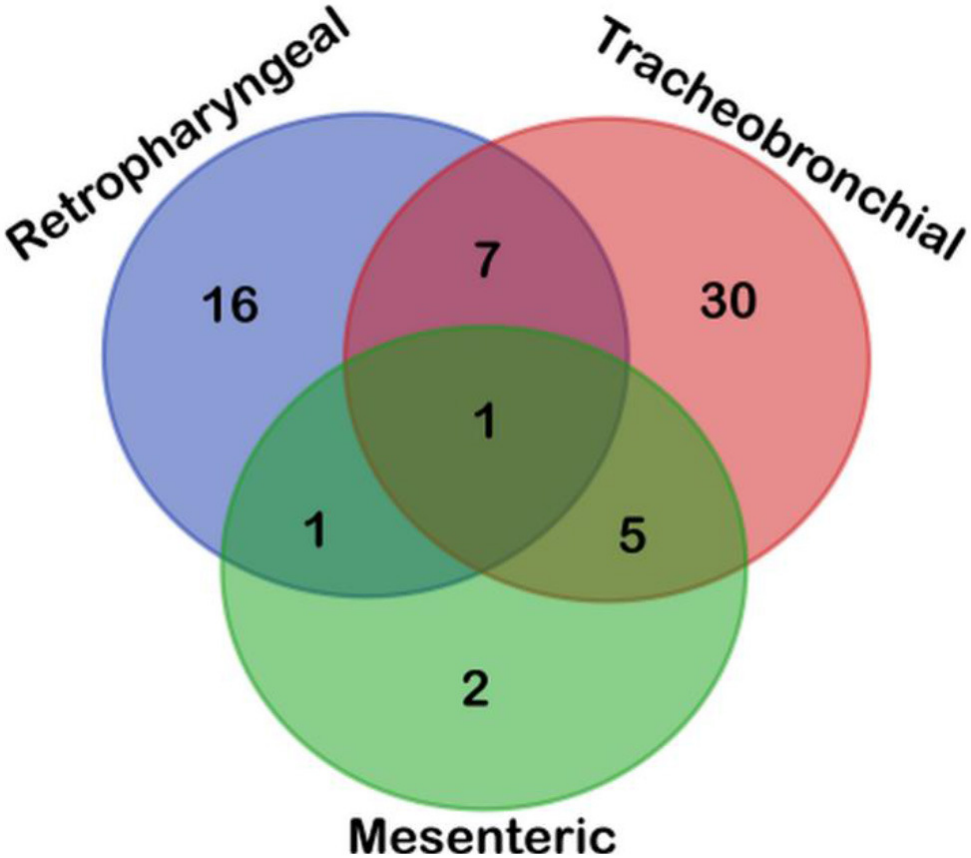
Venn diagram depicted the topographical distribution of positive lymph nodes (*n* = 77) according to their results obtained upon analyzing samples by microbiological culture, qPCR-IS*6110*, and Sanger sequencing. Most positive cattle were disclosed in tracheobronchial lymph node, followed by retropharyngeal and mesenteric lymph nodes, with most positive animals presenting only one positive lymph node highlighting the choice of tissue is a cornerstone for performing an accurate direct diagnosis of MTC.

Finally, according to the distribution of MTC-corrected positive results for the lymph nodes (77), the majority of MTC-corrected positive animals (48) were detected in only one lymph node (tracheobronchial = 30; retropharyngeal = 16; mesenteric = 2), 13 animals in two lymph nodes (retropharyngeal–tracheobronchial = 7; tracheobronchial–mesenteric = 5; retropharyngeal–mesenteric = 1), and one animal in the three lymph nodes, as showed in Figure 2 (Table 4).

**Table 4:** Assessment of the microbiological culture and direct qPCR targeting IS*6110* results obtained upon analyzing 687 lymph nodes belonging to 230 cattle, according to the presence or absence of tuberculosis-like lesions.

		**Microbiological culture**			
		**+**	**–**	**Total**	
NTBL	qPCR +	36	1	37	661
	qPCR −	17	607	624	
TBL	qPCR +	20	3	23	26
	qPCR −	0	3	3	

*+, Positive; –, Negative; qPCR, real-time PCR; 95% CI, 95% confidence level; NTBL, non-tuberculosis-like lesion; TBL, tuberculosis-like lesion*.

### Bacteriology and Quantitative Real-Time PCR Results Distribution According to Tuberculosis-Like Lesion

Analyzing MTC and qPCR results together with the presence of TBL, 26 of 687 tissue samples (3.8%) showed TBL, and 20 of these 26 samples (76.9%) resulted in both culture and qPCR positive. The remaining six were negative to culture, being three of them also negative to qPCR. In contrast, the other three were positive to qPCR and subsequently confirmed by Sanger sequencing.

Thirty-six of 661 NTBL tissue samples (5.4%) were positive to either microbiological or qPCR assays, 17 were only culture-positive (2.6%), and 1 only qPCR positive (0.2%). Of note, this culture-negative and qPCR-positive sample was confirmed as positive after Sanger sequencing. Thus, 607 NTBL tissue samples (91.8%) were negative for both techniques (Table 3).

## Discussion

bTB is one of the oldest and most relevant zoonoses worldwide, where its eradication is the main objective of the EU. As a consequence, rapid, cost-effective, and sensitive tools for the diagnosis of different pathogens belonging to MTC play a pivotal role in controlling and preventing its transmission in countries where it is still especially present in dairy and meat cattle herds (13, 15, 28). Therefore, direct qPCR from tissue samples could work as an accurate and rapid diagnostic alternative in animal health (7, 11, 13, 23), which could be implemented by public health agencies not only to reduce the turnaround time on reaching a confirmatory diagnosis compared with microbiological culture but also to shorten the time of exposure to MTC, facilitating the decision-making process. In this context, the main objective of the present study was to evaluate a qPCR targeting IS*6110* to detect MTC directly from fresh tissue bovine lymph node samples.

In the present study, the direct qPCR targeting IS*6110* showed an apparent SE and SP for individual tissue samples of 76.7 and 99.3%, respectively, when compared with microbiological culture. In addition, the agreement between both assays was almost perfect (κ = 0.83). Several factors make it challenging to run a direct detection of MTC, such as the paucibacillary nature of this complex, the extremely hardy disruption of mycobacterial cells, or the extensive necrosis, fibrosis, and mineralization associated with TBL, interfering all of them with mycobacterial DNA isolation and leading to false-negative results, which limits the final diagnosis performance (19, 29, 30). In our case, samples were individually homogenized before the process to reach a uniform distribution of MTC in the whole matrix but also trying to restrict a dilution effect beyond the detection limit of the qPCR. Despite that, 20 samples were positive to microbiological culture but negative for qPCR. It is well-known that the yield and quality of DNA after extraction could depend on multiple factors (11, 15); consequently, DNA isolation was repeated in all qPCR-negative samples increasing proteinase K digestion up to 12 h at 56°C (overnight incubation), obtaining three additional qPCR-positive samples, and slightly improving SE from 71.2 to 76.7%. A similar approach was conducted to improve diagnostic SE and SP of direct qPCR targeting mpb70 from 88.4 and 92.3 to 94.5 and 96.0%, respectively (11). These results suggest that DNA extraction protocol is certainly relevant, impacting directly on diagnostic SE of direct qPCR from fresh tissue samples.

Several alternative methods have been previously performed to improve diagnostic SE of direct qPCR from tissues. Thus, nested-PCR targeting TbD1 (24) or IS*6110* (17) have been suggested as a method to improve the detection of MTC in bovine tissue samples with a diagnostic SE and SP ranging from 76.0 to 98.2% and from 88.7 to 100%, respectively. Nevertheless, a nested-PCR requires two different amplification steps, increasing the concern about cross-contamination, which could negatively affect diagnostic SP values. On the other hand, Parra et al. (22) used a manual extraction method with capture probes targeting 16S-23S internally transcribed spacer region to isolate a higher yield of mycobacterial DNA from tissue homogenate samples obtaining a diagnostic SE ranging from 61.1% for samples with NTBL to 80.6% for TBL samples, with an average SE of 73.8%. In this sense, Taylor et al. (19) reported an increase of diagnostic SE from 70.1 to 91.2% targeting *IS*1081 and carrying out DNA isolation only from TBL, ruling out positive samples without readily macroscopic lesions. In our case, 23 of 26 lymph nodes with TBL (88.5%) were amplified targeting IS*6110*; nevertheless, when all samples were considered, both TBL and NTBL samples, an SE of 76.7% was obtained, highlighting the potential of using this target for qPCR screening not only in TBL but also in NTBL.

Previous studies targeting mpb70 (11) or IS*6110* (7) have reported higher SE and SP results than those herein reported; however, it is noteworthy to mention that in those studies, there was a high proportion of the evaluated samples with TBL (39.8 and 100%, respectively). This feature evidences that animals included in those studies were in more advanced stages of bTB infection (15, 31). Unlikely, in the present study, most of the tissue samples lacked TBL (661/687), with only 3.6% of them presenting TBL, which points to animals were sampled in earlier stages of the infection. In addition, qPCR targeting IS*6110* showed a moderate diagnostic SE and high SP. These results highlight the diagnostic potential of direct qPCR from fresh tissue to detect MTC at early stages of infection and, therefore, when the mycobacterial load is lower.

Regarding the topographical distribution of the lesions, most of the TBL samples were disclosed in the tracheobronchial lymph node, followed by retropharyngeal and mesenteric lymph nodes, with most positive animals presenting only one affected lymph node (77.4%). One of the strengths of the present study is that a detailed evaluation of the topographical distribution of the results was made, as the choice of tissue samples at the abattoir is a key player for carrying out an accurate direct diagnosis of MTC. In addition, the diagnosis from a pool of lymph nodes from reactor animals with TBL or NTBL is probably to have a dilution impact on the results. According to our results, most of the true positive animals reacted in one single lymph node, highlighting that not only tracheobronchial and retropharyngeal lymph nodes but also mesenteric lymph node, which is uncommonly sampled during postmortem inspection in bTB surveillance systems at the slaughterhouse, should be evaluated and collected for TB diagnosis. These results turn out to be relevant in areas with a low TB prevalence scenario to enhance the diagnostic accuracy of direct detection methods.

Although microbiological culture is considered the *gold standard* for bTB confirmation, this technique is time-consuming and imperfect, inducing false-negative results (8), and SE and SP will always be biased (7, 32); therefore, combination with other techniques is required to truly identify MTC positive samples. Thereby, three of six samples with TBL and culture-negative were detected as positive for direct qPCR and confirmed by Sanger sequencing, displaying a SE and SP of 77.9 and 100%, respectively, for MTC detection. It is worthy of note that the other three culture-negative and qPCR-negative TBL samples presented pyogranulomatous lesions and Ziehl–Neelsen negative results when examined under the light microscope (data not shown). These results suggest that other microorganisms may be involved in the production of these lesions, as has already been demonstrated in pigs (33), which could be taken into account for future studies.

Several factors impact the success of microbiological cultures, such as decontamination process (8) or encapsulation of the granulomas (34); however, DNA amplification of MTC can be successfully performed from fibrotic and encapsulated granulomas. Our results highlight that direct qPCR can detect more positive samples from fresh lymph nodes tissue with TBL than microbiological culture (23 vs. 20), resulting in a faster and effective confirmatory method for MTC during official postmortem inspection at the slaughterhouse. Nonetheless, microbiological culture remains a required method to mycobacterial isolation and molecular epidemiology studies so far (7).

Finally, direct qPCR targeting IS*6110* was also used to run the diagnostic performance of all animals included in this study, showing diagnostic SE and SP values of 77.0 and 99.4%, respectively, which are very close to those previously reported for microbiological culture (7). In addition, predictive values of 97.9% PPV and 92.3% NPV, together with PLR and NLR of 130.2 and 0.23, respectively, point to a qPCR-positive animal could be considered as true positive. Previous reports on animals have indicated either a barely higher SE for qPCR targeting IS*6110* (7) or lower SE for *IS*1081 (16) compared with the diagnostic SE herein reported. In addition, if diagnostic estimates for MTC detection were considered, SE and SP values could be increased up to 77.4 and 100%, respectively. The disparities among studies may be attributed to differences in the approach for data analysis, epidemiologic situation, or the sample size. On the other hand, gross postmortem examination is a critical stage for the detection of bTB-infected animals at slaughterhouses. Nevertheless, the number of reactors with TBL is currently reduced at the slaughterhouse due to the success of surveillance and control programs decreasing bTB prevalence in cattle herds. Therefore, in the present framework, qPCR assay targeting IS*6110* might work as a suitable complementary method to confirm bTB in reactor animals with either TBL or NTBL, decreasing the number of samples subjected to microbiological culture and, hence, the overall associated cost as well as the turnaround time, <48 h, for confirming bTB infection.

## Conclusion

The present study revealed that qPCR targeting IS*6110* is an efficient confirmatory test that may be implemented in bTB surveillance and control programs, shortening turnaround time to keep decision-makers noticed promptly, as well as reducing economic costs.

## Data Availability Statement

The original contributions presented in the study are included in the article/supplementary material, further inquiries can be directed to the corresponding author/s.

## Author Contributions

JS-C and ÁG-R performed all experiments in this study and Animal Health and Production Laboratory of Córdoba supported with microbiological culture and technical assistance. ÁG-R, JS-C, IR-T, EV, FJ-M, FL-M, FC-T, and LG-G were involved in obtaining and processing tissue samples. LC, IL, JG-L, and CT designed the study. JS-C and ÁG-R wrote the manuscript down with the invaluable insights of JG-L, IL, IR-T, IR-G, FJ-M, LG-G, FC-T, LC, CT, and AM. IL and JG-L directed and supervised the whole study. All authors contributed to the article and approved the submitted version.

## Conflict of Interest

The authors declare that the research was conducted in the absence of any commercial or financial relationships that could be construed as a potential conflict of interest.
